# Maternal exposure to smoking and wheezing phenotypes in children: a cohort study of the Japan Environment and Children’s Study

**DOI:** 10.1186/s12887-024-05101-6

**Published:** 2024-10-01

**Authors:** Takuya Wada, Yuichi Adachi, Shokei Murakami, Yasunori Ito, Toshiko Itazawa, Akiko Tsuchida, Kenta Matsumura, Kei Hamazaki, Hidekuni Inadera, on behalf of the Japan Environment and Children’s Study Group

**Affiliations:** 1 Department of Pediatrics, Faculty of Medicine, University of Toyama, Toyama, Japan; 2 Pediatric Allergy Center, Toyama Red Cross Hospital, 2-1-58 Ushijima-honmachi, Toyama, 930- 8562 Japan; 3 Department of Pediatrics, Saitama Medical University, Saitama, Japan; 4 Department of Public Health, Faculty of Medicine, University of Toyama, Toyama, Japan; 5 Toyama Regional Center for Japan Environment and Children’s Study (JECS), University of Toyama, Toyama, Japan; 6 Department of Public Health, Gunma University Graduate School of Medicine, Gunma, Japan

**Keywords:** Asthma, Birth cohort, Respiratory sounds, Secondhand smoke, Tobacco smoking, Wheeze trajectories

## Abstract

**Background:**

Previous studies have shown that prenatal maternal smoking and maternal secondhand smoke exposure during pregnancy were associated with an increased risk of wheezing and asthma development. However, few studies have examined the influence of different sources of tobacco exposure in different perinatal timeframes (preconception, prenatal, and postnatal) on wheezing phenotypes in children. Using national survey data from Japan, we investigated the effects of exposure to tobacco smoke during pregnancy on wheezing phenotypes in children before the age of 3 years.

**Methods:**

Pregnant women who lived in the 15 regional centers in the Japan Environment and Children’s Study were recruited. We obtained information on prenatal and postnatal exposure to active and secondhand smoke (SHS) and wheeze development up to 3 years of age. Multiple logistic regression analysis was performed to determine the association between tobacco smoke exposure and wheezing phenotypes in children.

**Results:**

We analyzed 73,057 singleton births and identified four longitudinal wheezing phenotypes: never wheezing; early transient wheezing (wheezing by age 1 year but not thereafter); late-onset wheezing (wheezing by age 2–3 years but not beforehand); and persistent wheezing. Maternal smoking during pregnancy was significantly associated with early transient and persistent wheezing in children compared with no maternal smoking [early transient wheezing: 1–10 cigarettes per day, adjusted odds ratio (aOR) 1.43, 95% confidence interval (CI) 1.23–1.66; ≥ 11 cigarettes per day, aOR 1.67, 95% CI 1.27–2.20; persistent wheezing: 1–10 cigarettes per day, aOR 1.64, 95% CI 1.37–1.97; ≥ 11 cigarettes per day, aOR 2.32, 95% CI 1.70–3.19]. Smoking cessation even before pregnancy was also significantly associated with increased risk of early transient wheezing, late-onset wheezing, and persistent wheezing in children. Moreover, maternal exposure to SHS during pregnancy was significantly associated with increased risk of early transient and persistent wheezing compared with no such exposure.

**Conclusions:**

Maternal smoking before and throughout pregnancy was associated with wheeze development in children up to 3 years of age. It appears that smoking is detrimental compared to never smoking, regardless of whether individuals quit smoking before or after becoming aware of the pregnancy.

**Supplementary Information:**

The online version contains supplementary material available at 10.1186/s12887-024-05101-6.

## Background

The emerging concept of the Developmental Origins of Health and Disease (DOHaD) hypothesis posits that exposure of the embryo or fetus to various environmental toxins can contribute to the development of non-communicable diseases in childhood [1]. Furthermore, exposure to environmental factors, such as smoking, drinking, and nutrition, through the mother during the embryonic period has been reported to affect the development of childhood allergic disease [2]. In particular, the prenatal and early postnatal periods have been identified as key timeframes in which specific environmental exposures increase susceptibility to later asthma development [3]. Tobacco smoking is an environmental factor that can be prevented, and it is important to investigate tobacco smoke exposure in the perinatal period and the subsequent development of wheezing and asthma in children.

In a previous meta-analysis, prenatal maternal smoking was associated with an increased risk of wheezing development in the first 12 months of life [4]. Maternal secondhand smoke (SHS) exposure during pregnancy was also found to be an independent risk factor for wheezing in children up to the age of 2 years [5]. Similarly, we previously showed that current maternal smoking and maternal exposure to SHS during pregnancy significantly increased the risk of wheezing and asthma in the first year of life [6].

Wheezing is a crucial determinant of the development of asthma. Within the last decade, there has been a surge of data describing various methods for identifying wheezing phenotypes in childhood [7]. Indeed, there is growing recognition that the various phenotypes of childhood wheezing and the identification of associated risk factors are critical for developing targeted preventive and therapeutic strategies for childhood asthma [8]. A recent systematic review and meta-analysis with a follow‐up duration ranging from 3 to 18 years identified five groups based on the onset and duration of childhood wheezing episode (never/infrequent, early transient, early persistent, intermediate-onset, and late-onset) and showed that tobacco exposure, irrespective of definition (prenatal vs postnatal, maternal vs paternal, duration or dose), was associated with early transient, early persistent, and late-onset wheeze but not intermediate-onset wheeze [9]. There is limited evidence, however, regarding the influence of different sources of tobacco smoke exposure (maternal active smoking and SHS) in different timeframes (preconception, prenatal, and postnatal periods) on the risk of wheezing phenotypes in children. In this study, using data from a nationwide birth cohort study in Japan, we investigated the effects of exposure to tobacco smoke from preconception to postnatal period on wheezing phenotypes in children before the age of 3 years.

## Methods

### Study design and oversight

The Japan Environment and Children’s Study (JECS) is a birth cohort study of 100,000 pairs of children and their mothers throughout Japan. It has been conducted since 2011, with pregnant women recruited between January 2011 and March 2014. The JECS aims to identify the environmental factors that affect children’s health and development. The background and general procedures of the JECS are described in detail elsewhere [10, 11]. The JECS protocol was reviewed and approved by the Institutional Review Board of the Ministry of Environment on Epidemiological Studies and the ethics committees of all participating institutions. Written informed consent was obtained from all participants.

### Study participants

The eligibility criteria for participating in the JECS were as follows: 1) residence in one of the study areas at the time of recruitment, 2) expected birth date between August 2011 and mid-2014, and 3) able to understand the Japanese language and complete self-administered questionnaires. We excluded pregnant women who resided outside the study areas but attended co-operating health care providers within the areas.

**Fig. 1:**
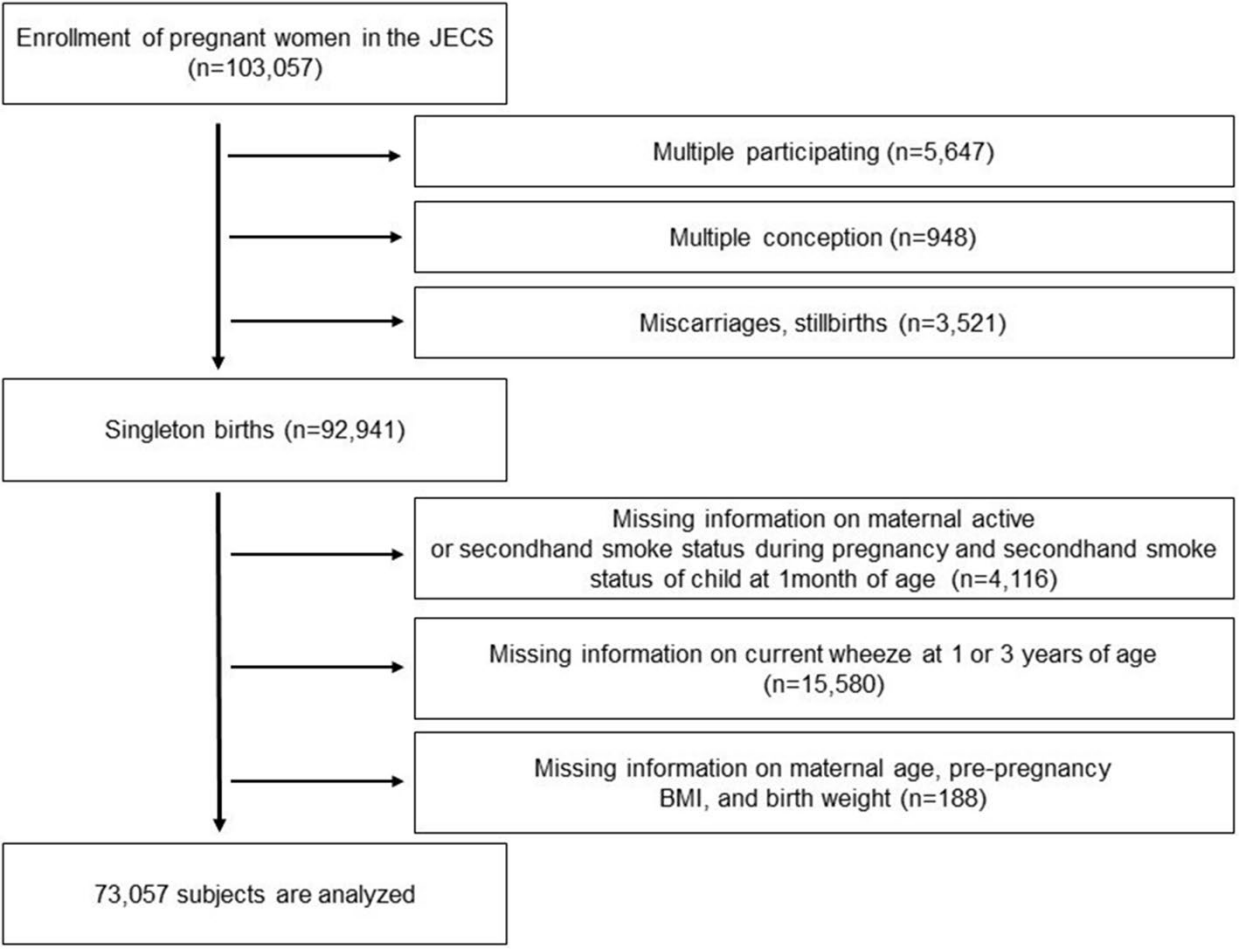
Flow diagram of the recruitment process. Abbreviation: BMI, body mass index

The dataset analyzed in the present study was the jecs-qa-20210401 (jecs-ta-20190930) dataset that was released in April 2021. It contains 103,057 pregnancies, after the exclusion of mothers who withdrew their consent. From these 103,057 pregnancies, 92,941 mother–infant pairs were identified. After excluding participants with missing information on prenatal or postnatal exposure to active smoking or SHS, current wheeze at 1 or 3 years of age, maternal age, prepregnancy body mass index, or birth weight, we analyzed 73,057 singleton births (Fig. 1).

### Data collection

Mothers completed self-administered questionnaires distributed during the first and second/third trimesters. Fathers’ information was also collected using a self-administered questionnaire during the pregnancy. Children were followed up through self-administered questionnaires completed by their mothers or caregivers at 1 month and 6 months of age and then every 6 months up to 3 years of age. The questionnaire collected information on demographic factors, medical history, delivery information, lifestyle, socioeconomic status, and environmental exposure. In addition, perinatal information and infants’ physical examination findings were obtained from the medical records [12, 13].

### Assessment of smoking habits

Self-administered questionnaires about maternal smoking status during pregnancy were distributed 1 month postpartum. Maternal smoking status during pregnancy was evaluated using the following question: “Please choose an answer regarding your smoking history” (1 = never smoked; 2 = previously smoked, but quit before learning of the pregnancy; 3 = previously smoked, but quit after learning of the pregnancy; 4 = currently smoke). To avoid small numbers of participants, the data for current smoker were recategorized into 1–10 and ≥ 11 cigarettes/day.

Prenatal maternal exposure to SHS was evaluated using a questionnaire administered during the second/third trimester of pregnancy. Postnatal exposure of the infant to SHS was assessed at 1 month postpartum. Prenatal maternal exposure to SHS was assessed as the number of days per week of exposure to tobacco smoke at home, in the workplace, or in other indoor locations. Postnatal exposure of the infant to SHS was established by asking about the location of such exposure (none, outdoor, or indoor). The questionnaire did not ask about the type of tobacco product consumed (combusted or heated).

### Outcome measures

The primary outcome measure for this study was the incidence of wheezing phenotypes in children up to 3 years of age. The presence of wheezing episodes was assessed using a self-administered questionnaire completed by the mother or caregiver when the child was 1 and 3 years old. Current wheeze was defined as a positive response to the following question, based on International Study of Asthma and Allergies in Childhood questionnaires: “Has your child ever had wheezing or whistling in the chest in the last 12 months?” [14–16]. The children were assigned into the following four groups according to their history of wheezing with reference to the report of Martinez et al. [17]: 1) never wheezing—those who had no recorded lower respiratory tract illness with wheezing by 1 year of age and had no wheezing by 2–3 years of age; 2) early transient wheezing—those with at least one lower respiratory tract illness with wheezing by 1 year of age but no wheezing by 2–3 years of age; 3) late-onset wheezing—those who had no lower respiratory tract illness with wheezing by 1 year of age but who had wheezing by 2–3 years of age; and 4) persistent wheezing—those who had at least one lower respiratory tract illness with wheezing by 1 year of age and had wheezing by 2–3 years of age.

### Covariates

The following variables were assessed using a self-administered questionnaire and included in the analysis as covariates according to previous reports: exposure of the pregnant mothers and children to tobacco smoke; demographic variables (maternal employment status, maternal education level, annual household income); lifestyle factors (maternal physical activity, maternal alcohol consumption); and medical history (maternal age at delivery, maternal history of allergy, mode of delivery). Maternal history of allergy (asthma, atopic dermatitis, food allergy, allergic rhinitis, or allergic conjunctivitis) based on a physician’s diagnosis was confirmed using a self-administered questionnaire. The child’s history of allergic disease was assessed at 1, 1.6, 2, and 3 years of age, respectively, using self-administered questionnaires. This study specified 31 congenital anomalies that are easily detectable at birth and require prompt treatment [18]. For all variables, the questionnaire content, respondent group and response period are summarized in Supplementary Table 1.

### Statistical analysis

Multiple logistic regression analysis was conducted to identify the association between tobacco smoke exposure in pregnant mothers and each wheezing phenotype (ie, never wheezing, early transient wheezing, late-onset wheezing, and persistent wheezing) in their children by 3 years of age. Three sets of regression analyses were conducted taking the dependent variables as follows: (1) never wheezing vs early transient wheezing, (2) never wheezing vs late-onset wheezing and (3) never wheezing vs persistent wheezing.

This analysis was used to calculate crude odds ratio (OR) and adjusted OR (aOR) and 95% confidence intervals (CI). The regression model was adjusted for maternal age at delivery, maternal body mass index before pregnancy, maternal physical activity during mid-late pregnancy, marital status, maternal employment status, maternal education level, maternal alcohol consumption, maternal history of allergy, infant sex, gestational weeks at delivery, birth weight, mode of delivery, birth season, parity, infant anomalies, daycare attendance, pet ownership (any kind of animal), and annual household income. The Bonferroni correction was applied for multiple comparisons, and the significance level was set at *p* < 0.017. Missing data were also included in the model as dummy-coded variables. In the trend test, each category in each smoking variable was assigned a numerical value starting from 1 and then rated as a continuous variable.

All analyses were performed using SAS version 9.4 (SAS Institute Inc., Cary, NC, USA).

## Results

Tables 1, 2, 3 and 4 show the characteristics of the participants. Of the mothers who reported smoking status during pregnancy, 44,561 (61.0%) were never smokers, 16,567 (22.7%) quit smoking before learning of the pregnancy, 9595 (13.1%) quit smoking after learning of the pregnancy, 1890 (2.6%) were current smokers of 1–10 cigarettes per day, and 444 (0.6%) were current smokers of ≥ 11 cigarettes per day, respectively. The percentage of mothers who reported exposure to SHS at least once a week during pregnancy was 26,111(35.7%). Postnatal exposure of the infant to SHS, without considering the location, was 36,648(50.2%). The prevalence of maternal allergic diseases, based on a physician’s diagnosis, was 48.1%. Among the 73,057 children, 54,853 (75.1%) had the never wheezing phenotype. Of the children who had wheezing, 6983 (9.6%) were classified as having early transient wheezing, 7201 (9.9%) had late-onset wheezing, and 4020 (5.5%) had persistent wheezing. Atopic dermatitis was the most common allergic disease when the child was 3 years old (Supplementary Table 2).

**Table 1:** Wheezing phenotypes in children according to prenatal and postnatal exposure to tobacco smoke

			Wheezing phenotypes in offspring during the first 3 years of life							
	All		Never wheezing		Early transient wheezing		Late-onset wheezing		Persistent wheezing	
	73,057	(100%)	54,853	(75.1%)	6983	(9.6%)	7201	(9.9%)	4020	(5.5%)
Maternal smoking status at 1 month postpartum										
Never smoker	44,561	(61.0%)	34,272	(62.5%)	3839	(55.0%)	4282	(59.5%)	2168	(53.9%)
Quit smoking before learning of the pregnancy	16,567	(22.7%)	12,148	(22.2%)	1734	(24.8%)	1684	(23.4%)	1001	(24.9%)
Quit smoking after learning of the pregnancy	9595	(13.1%)	6938	(12.7%)	1054	(15.1%)	990	(13.8%)	613	(15.3%)
Current smoker during pregnancy										
1–10 cigarettes/day	1890	(2.6%)	1224	(2.2%)	282	(4.0%)	201	(2.8%)	183	(4.6%)
≥ 11 cigarettes/day	444	(0.6%)	271	(0.5%)	74	(1.1%)	44	(0.6%)	55	(1.4%)
Frequency of SHS exposure in second/third trimester										
Almost never	46,946	(64.3%)	36,096	(65.8%)	4056	(58.1%)	4515	(62.7%)	2279	(56.7%)
≤ 1 day per week	8784	(12.0%)	6438	(11.7%)	922	(13.2%)	905	(12.6%)	519	(12.9%)
2–3 days per week	5697	(7.8%)	4152	(7.6%)	611	(8.8%)	566	(7.9%)	368	(9.2%)
4–6 days per week	3467	(4.7%)	2452	(4.5%)	411	(5.9%)	374	(5.2%)	230	(5.7%)
Every day	8163	(11.2%)	5715	(10.4%)	983	(14.1%)	841	(11.7%)	624	(15.5%)
Location of exposure to tobacco smoke at 1 month postpartum										
None	36,409	(49.8%)	28,071	(51.2%)	3098	(44.4%)	3516	(48.8%)	1724	(42.9%)
Outdoor	35,062	(48.0%)	25,694	(46.8%)	3675	(52.6%)	3529	(49.0%)	2164	(53.8%)
Indoor	1586	(2.2%)	1088	(2.0%)	210	(3.0%)	156	(2.2%)	132	(3.3%)

*Abbreviations*: *SHS* Secondhand smoke, *SD* Standard deviation

**Table 2:** Characteristics of mothers in relation to child’s wheezing phenotype

	Child’s wheezing phenotype during first 3 years of life			
	Never wheezing	Early transient wheezing	Late-onset wheezing	Persistent wheezing
	54,853 (75.1%)	6983 (9.6%)	7201 (9.9%)	4020 (5.5%)
Maternal age at delivery, years				
< 25	4711 (8.6%)	688 (9.9%)	623 (8.7%)	342 (8.5%)
25– < 30	15,234 (27.8%)	2013 (28.8%)	2066 (28.7%)	1143 (28.4%)
30– < 35	19,750 (36.0%)	2522 (36.1%)	2596 (36.1%)	1513 (37.6%)
≥ 35	15,158 (27.6%)	1760 (25.2%)	1916 (26.6%)	1022 (25.4%)
Maternal BMI before pregnancy, kg/m2				
< 18.5	9041 (16.5%)	1062 (15.2%)	1130 (15.7%)	601 (15.0%)
18.5–24.9	40,619 (74.1%)	5199 (74.5%)	5342 (74.2%)	2939 (73.1%)
≥ 25	5193 (9.5%)	722 (10.3%)	729 (10.1%)	480 (11.9%)
Physical activity during mid-late pregnancy				
No	13,021 (23.7%)	1662 (23.8%)	1724 (23.9%)	956 (23.8%)
Yes	41,749 (76.1%)	5307 (76.0%)	5463 (75.9%)	3055 (76.0%)
Missing data	83 (0.2%)	14 (0.2%)	14 (0.2%)	9 (0.2%)
Marital status				
Married	52,323 (95.4%)	6646 (95.2%)	6878 (95.5%)	3858 (96.0%)
Single	1821 (3.3%)	219 (3.1%)	213 (3.0%)	106 (2.6%)
Widowed, divorced	325 (0.6%)	73 (1.1%)	55 (0.8%)	35 (0.9%)
Missing data	384 (0.7%)	45 (0.6%)	55 (0.8%)	21 (0.5%)
Maternal education, years				
≤ 12 years	18,038 (32.9%)	2604 (37.3%)	2240 (31.1%)	1395 (34.7%)
13–15 years	23,303 (42.5%)	3092 (44.3%)	3191 (44.3%)	1850 (46.0%)
≥ 16 years	13,275 (24.2%)	1254 (18.0%)	1744 (24.2%)	754 (18.8%)
Missing data	237 (0.4%)	33 (0.5%)	26 (0.4%)	21 (0.5%)

*Abbreviation*: *BMI* Body mass index

**Table 3:** Characteristics of mothers in relation to child’s wheezing phenotype

	Wheezing phenotypes in offspring during the first 3 years of life			
	Never wheezing	Early transient wheezing	Late-onset wheezing	Persistent wheezing
	54,853 (75.1%)	6983 (9.6%)	7201 (9.9%)	4020 (5.5%)
Maternal employment status				
No	25,652 (46.8%)	2824 (40.4%)	3022 (42.0%)	1483 (36.9%)
Yes	28,777 (52.5%)	4112 (58.9%)	4123 (57.3%)	2506 (62.3%)
Missing data	424 (0.8%)	47 (0.7%)	56 (0.8%)	31 (0.8%)
Maternal alcohol consumption				
Never	18,727 (34.1%)	2224 (31.9%)	2300 (31.9%)	1256 (31.2%)
Former drinker	34,370 (62.7%)	4461 (63.9%)	4672 (64.9%)	2599 (64.7%)
Current drinker	1372 (2.5%)	250 (3.6%)	181 (2.5%)	140 (3.5%)
Missing data	384 (0.7%)	48 (0.7%)	48 (0.7%)	25 (0.6%)
Maternal history of allergy				
No	28,299 (51.6%)	3238 (46.4%)	3061 (42.5%)	1571 (39.1%)
Yes	26,369 (48.1%)	3723 (53.3%)	4108 (57.1%)	2440 (60.7%)
Missing data	185 (0.3%)	22 (0.3%)	32 (0.4%)	9 (0.2%)
Asthma	4555 (8.3%)	982 (14.1%)	1219 (17.0%)	900 (22.4%)
Atopic dermatitis	8416 (15.4%)	1153 (16.6%)	1327 (18.5%)	762 (19.0%)
Food allergy	2335 (4.3%)	378 (5.4%)	457 (6.4%)	288 (7.2%)
Allergic rhinitis	19,238 (35.2%)	2692 (38.7%)	2993 (41.8%)	1774 (44.2%)
Allergic conjunctivitis	5116 (9.4%)	755 (10.9%)	961 (13.4%)	603 (15.0%)

**Table 4:** Characteristics of children and maternal lifestyle factors and socioeconomic status in relation to child’s wheezing phenotype

	Wheezing phenotypes in offspring during the first 3 years of life			
	Never wheezing	Early transient wheezing	Late-onset wheezing	Persistent wheezing
	54,853 (75.1%)	6983 (9.6%)	7201 (9.9%)	4020 (5.5%)
Child’s characteristics				
Male infant	26,839 (48.9%)	4029 (57.7%)	4050 (56.2%)	2514 (62.5%)
Gestational weeks				
< 34	367 (0.7%)	60 (0.9%)	77 (1.1%)	55 (1.4%)
34–36	1830 (3.3%)	246 (3.5%)	312 (4.3%)	202 (5.0%)
37–40	47,191 (86.0%)	6118 (87.6%)	6135 (85.2%)	3478 (86.5%)
≥ 41	5465 (10.0%)	559 (8.0%)	677 (9.4%)	285 (7.1%)
Birth weight, < 2500 g				
No	50,632 (92.3%)	6468 (92.6%)	6584 (91.4%)	3681 (91.6%)
Yes	4221 (7.7%)	515 (7.4%)	617 (8.6%)	339 (8.4%)
Type of delivery, Caesarean				
No	44,762 (81.6%)	5705 (81.7%)	5780 (80.3%)	3200 (79.6%)
Yes	9985 (18.2%)	1263 (18.1%)	1408 (19.6%)	810 (20.2%)
Missing data	106 (0.2%)	15 (0.2%)	13 (0.2%)	10 (0.3%)
Birth season				
March–May	12,828 (23.4%)	1629 (23.3%)	1588 (22.1%)	916 (22.8%)
June–August	14,750 (26.9%)	1969 (28.2%)	1761 (24.5%)	985 (24.5%)
September–November	15,058 (27.5%)	1876 (26.9%)	2067 (28.7%)	1130 (28.1%)
December–February	12,217 (22.3%)	1509 (21.6%)	1785 (24.8%)	989 (24.6%)
Parity				
0	24,999 (45.6%)	2246 (32.2%)	3058 (42.5%)	1102 (27.4%)
≥ 1	28,386 (51.8%)	4606 (66.0%)	3955 (54.9%)	2851 (70.9%)
Missing data	1468 (2.7%)	131 (1.9%)	188 (2.6%)	67 (1.7%)
Infant anomalies				
No	53,699 (97.9%)	6820 (97.7%)	7021 (97.5%)	3909 (97.2%)
Yes	1154 (2.1%)	163 (2.3%)	180 (2.5%)	111 (2.8%)
Daycare attendance				
No	51,840 (94.5%)	5867 (84.0%)	6790 (94.3%)	3328 (82.8%)
Yes	2569 (4.7%)	1027 (14.7%)	354 (4.9%)	649 (16.1%)
Missing data	444 (0.8%)	89 (1.3%)	57 (0.8%)	43 (1.1%)
Pet ownership				
No	41,355 (75.4%)	5173 (74.1%)	5229 (72.6%)	2992 (74.4%)
Yes	12,951 (23.6%)	1709 (24.5%)	1908 (26.5%)	977 (24.3%)
Missing data	547 (1.0%)	101 (1.5%)	64 (0.9%)	51 (1.3%)
Household income, million yen/year				
< 4	19,491 (35.5%)	2739 (39.2%)	2591 (36.0%)	1547 (38.5%)
4–6	17,363 (31.7%)	2123 (30.4%)	2261 (31.4%)	1245 (31.0%)
≥ 6	14,459 (26.4%)	1703 (24.4%)	1913 (26.6%)	979 (24.4%)
Missing data	3540 (6.5%)	418 (6.0%)	436 (6.1%)	249 (6.2%)

Tables 5 and 6 show the crude OR and aOR for child’s wheezing phenotypes associated with tobacco smoke exposure in pregnancy. In the crude model, prenatal maternal smoking status, except for current smokers who smoked ≥ 11 cigarettes per day, was associated with early transient, late-onset, and persistent wheezing. Moreover, similar associations were observed for maternal exposure to SHS in the second/third trimester and postnatal exposure of the infant to SHS.

**Table 5:** Associations between tobacco smoke exposure and wheezing phenotypes (crude models)

	Child’s wheezing phenotype during the first 3 years of life								
	Early transient wheezing			Late-onset wheezing			Persistent wheezing		
	Cases/Subtotal	cOR (95% CI)	*p*	Cases/Subtotal	cOR (95% CI)	*p*	Cases/Subtotal	cOR (95% CI)	*p*
Maternal smoking status at 1 month postpartum									
Never smoker	3839/38111	1		4282/38554	1		2168/36440	1	
Quit smoking before learning of pregnancy	1734/13882	1.27 (1.20–1.35)	< 0.001	1684/13832	1.11 (1.05–1.18)	< 0.001	1001/13149	1.30 (1.21–1.41)	< 0.001
Quit smoking after learning of pregnancy	1054/7992	1.36 (1.26–1.46)	< 0.001	990/7928	1.14 (1.06–1.23)	< 0.001	613/7551	1.40 (1.27–1.53)	< 0.001
Current smoker during pregnancy									
1–10 cigarettes/day	282/1506	2.06 (1.80–2.35)	< 0.001	201/1425	1.32 (1.13–1.53)	< 0.001	183/1407	2.36 (2.01–2.78)	< 0.001
≥ 11 cigarettes/day	74/345	2.44 (1.88–3.16)	< 0.001	44/315	1.30 (0.94–1.79)	0.109	55/326	3.21 (2.39–4.30)	< 0.001
* p* value for trend			< 0.001			< 0.001			< 0.001
Frequency of SHS exposure in second/third trimester									
Almost never	4056/40152	1		4515/40611	1		2279/38375	1	
≤ 1 day per week	922/7360	1.28 (1.18–1.38)	< 0.001	905/7343	1.12(1.04–1.21)	0.003	519/6957	1.28(1.16–1.41)	< 0.001
2–3 days per week	611/4763	1.31 (1.20–1.43)	< 0.001	566/4718	1.09(0.99–1.20)	0.070	368/4520	1.40(1.25–1.57)	< 0.001
4–6 days per week	411/2863	1.49 (1.33–1.66)	< 0.001	374/2826	1.22(1.09–1.37)	< 0.001	230/2682	1.49(1.29–1.71)	< 0.001
Every day	983/6698	1.53 (1.42–1.65)	< 0.001	841/6556	1.18(1.09–1.27)	< 0.001	624/6339	1.73(1.58–1.90)	< 0.001
* p* value for trend			< 0.001			< 0.001			< 0.001
Location of exposure to tobacco smoke at 1 month postpartum									
None	3098/31169	1		3516/31587	1		1724/29795	1	
Outdoor	3675/29369	1.30 1.23–1.36)	< 0.001	3529/29223	1.10(1.04–1.15)	< 0.001	2164/27858	1.37(1.28–1.46)	< 0.001
Indoor	210/1298	1.75 (1.50–2.04)	< 0.001	156/1244	1.15(0.96–1.36)	0.122	132/1220	1.98(1.64–2.38)	< 0.001
* p* value for trend			< 0.001			< 0.001			< 0.001

*Abbreviations*: *CI* Confidence interval, *cOR* Crude odds ratio, *SHS* Secondhand smoke

**Table 6:** Associations between tobacco smoke exposure and wheezing phenotypes (adjusted models)

	Child’s wheezing phenotype during the first 3 years of life								
	Early transient wheezing			Late-onset wheezing			Persistent wheezing		
	Cases/Subtotal	aOR^a^ (95% CI)	*p*	Cases/Subtotal	aOR^a^ (95% CI)	*p*	Cases/Subtotal	aOR^a^ (95% CI)	*p*
Maternal smoking status at 1 month postpartum									
Never smoker	3839/38111	1		4282/38554	1		2168/36440	1	
Quit smoking before learning of pregnancy	1734/13882	1.12 (1.06–1.20)	< 0.001	1684/13832	1.08 (1.02–1.15)	0.012	1001/13149	1.13 (1.04–1.23)	0.003
Quit smoking after learning of pregnancy	1054/7992	1.19 (1.10–1.29)	< 0.001	990/7928	1.12 (1.03–1.21)	0.006	613/7551	1.24 (1.11–1.37)	< 0.001
Current smoker during pregnancy									
1–10 cigarettes/day	282/1506	1.43 (1.23–1.66)	< 0.001	201/1425	1.26 (1.07–1.49)	0.005	183/1407	1.64 (1.37–1.97)	< 0.001
≥ 11 cigarettes/day	74/345	1.67 (1.27–2.20)	< 0.001	44/315	1.28 (0.92–1.78)	0.145	55/326	2.32 (1.70–3.19)	< 0.001
* p* value for trend			< 0.001			< 0.001			< 0.001
Frequency of SHS exposure in second/third trimester									
Almost never	4056/40152	1		4515/40611	1		2279/38375	1	
≤ 1 day per week	922/7360	1.19 (1.10–1.29)	< 0.001	905/7343	1.09 (1.01–1.18)	0.030	519/6957	1.17 (1.06–1.30)	0.002
2–3 days per week	611/4763	1.13 (1.03–1.24)	0.014	566/4718	1.04 (0.95–1.15)	0.413	368/4520	1.20 (1.07–1.36)	0.003
4–6 days per week	411/2863	1.20 (1.07–1.35)	0.002	374/2826	1.14 (1.01–1.28)	0.032	230/2682	1.16 (1.00–1.35)	0.048
Every day	983/6698	1.14 (1.04–1.24)	0.005	841/6556	1.10 (1.00–1.20)	0.043	624/6339	1.24 (1.11–1.39)	< 0.001
* p* value for trend			< 0.001			0.010			< 0.001
Location of exposure to tobacco smoke at 1 month postpartum									
None	3098/31169	1		3516/31587	1		1724/29795	1	
Outdoor	3675/29369	1.09 (1.03–1.15)	0.005	3529/29223	1.05 (0.99–1.10)	0.115	2164/27858	1.15 (1.06–1.23)	< 0.001
Indoor	210/1298	1.15 (0.97–1.35)	0.108	156/1244	1.03 (0.86–1.23)	0.781	132/1220	1.24 (1.01–1.52)	0.045
* p* value for trend			0.003			0.151			< 0.001

*Abbreviations*: *aOR* Adjusted odds ratio, *CI* Confidence interval, *SHS* Secondhand smoke

^a^Adjusted for maternal age at delivery, maternal body mass index before pregnancy, maternal physical activity during mid-late pregnancy, marital status, maternal employment status, maternal education level, maternal alcohol consumption, maternal history of allergy, infant sex, gestational weeks at delivery, birth weight, mode of delivery, birth season, parity, infant anomalies, daycare attendance, pet ownership, and annual household income

Maternal smoking during pregnancy was associated with a high risk of early transient wheezing and persistent wheezing compared with no maternal smoking, with aOR of 1.43 (1–10 cigarettes per day, 95% CI 1.23–1.66) and 1.67 (≥ 11 cigarettes per day, 95% CI 1.27–2.20) for early transient wheezing and 1.64 (1–10 cigarettes per day, 95% CI 1.37–1.97) and 2.32 (≥ 11 cigarettes per day, 95% CI 1.70–3.19) for persistent wheezing. Mothers who smoked ≥ 11 cigarettes per day while pregnant had the greatest aOR value for both early transient wheezing and persistent wheezing. A higher risk tendency was observed for persistent wheezing than early transient wheezing. In a comparison of these phenotypes, maternal smoking during pregnancy was significantly associated with late-onset wheezing compared with no maternal smoking (1–10 cigarettes per day: aOR 1.26, 95% CI 1.07–1.49), although not in a dose-dependent manner (≥ 11 cigarettes per day: aOR 1.28, 95% CI 0.92–1.78). Quitting smoking after learning of the pregnancy was associated with a significantly increased risk of early transient wheezing (aOR 1.19, 95% CI 1.10–1.29) and persistent wheezing (aOR 1.24, 95% CI 1.11–1.37). In addition, quitting smoking before the pregnancy was significantly associated with increased risk of early transient wheezing (aOR 1.12, 95% CI 1.06–1.20), late-onset wheezing (aOR 1.08, 95% CI 1.02–1.15), and persistent wheezing (aOR 1.13, 95% CI 1.04–1.23). Similar results were obtained for maternal smoking during pregnancy when the analysis was performed only among children with no history of allergic disease (Supplementary Table 3).

Maternal exposure to SHS in the second/third trimester was associated with a significant increase in the incidence of early transient and persistent wheezing compared to no maternal exposure to SHS. For early transient wheezing, 4–6 days per week of maternal exposure to SHS had the highest aOR (aOR 1.20, 95% CI 1.07–1.35). For persistent wheezing, daily maternal exposure to SHS had the highest aOR (aOR 1.24, 95% CI 1.11–1.39).

Postnatal exposure of the infant to tobacco smoke outdoors was significantly associated with early transient wheezing (aOR 1.09, 95% CI 1.03–1.15) and persistent wheezing (aOR 1.15, 95% CI 1.06–1.23). However, children exposed to tobacco smoke indoors at 1 month postpartum exhibited no significant increased risk of any wheezing phenotype.

## Discussion

In this study of national survey data from Japan, we found that maternal smoking during pregnancy was significantly and dose-dependently associated with early transient and persistent wheezing in children up to 3 years of age compared with no maternal smoking. Furthermore, smoking cessation in early pregnancy was significantly associated with increased risk of early transient wheezing and persistent wheezing. Smoking cessation even before pregnancy was also significantly associated with increased risk of early transient wheezing, late-onset wheezing, and persistent wheezing.

Consistent with our results, a prospective birth cohort study in the Netherlands showed that maternal smoking during pregnancy was significantly associated with transient early wheeze, characterized by approximately 69% prevalence of wheezing at 12 months and declining prevalence thereafter, reaching 5% and 7% at 84 and 96 months, respectively [19, 20]. Furthermore, a prospective birth cohort study in the United Kingdom showed that maternal smoking during pregnancy was significantly associated with children’s wheezing at 3 years of age [21]. In a pooled analysis of eight European birth cohorts, maternal smoking during pregnancy was significantly associated with wheezing and asthma in preschool children [22]. The likelihood of developing wheezing and asthma increased significantly in a linear dose-dependent manner, particularly for smoking during the first trimester of pregnancy. Similar to these results, our study showed a significantly increased risk of early transient and persistent wheezing, even when mothers quit smoking after learning of their pregnancy (usually in the first trimester). The period of exposure to tobacco smoke in utero and the number of cigarettes smoked might be essential factors underlying the development of wheeze in children.

Emerging evidence suggests that mothers’ exposure to tobacco smoke before becoming pregnant may also impair their children’s respiratory health [23]. Epigenetics, particularly methylation changes, has been suggested as a potential mechanism involved in the pathogenesis of tobacco smoke-related diseases [24]. Epigenetic changes due to tobacco smoke may pass across generations through germ lines [25]. In this respect, we found that smoking cessation even before pregnancy still had a significant effect on the risk of early transient wheezing, late-onset wheezing, and persistent wheezing in children. Consistent with our results, a three-generation cohort study suggested that mothers’ exposure to smoking before their pregnancy was associated with an increased risk of asthma and lower lung function in their children [26–28]. Therefore, more attention should be paid to the fact that early smoking cessation by mothers improves health for future generations.

In the present study, maternal SHS exposure in the second/third trimester was significantly associated with early transient and persistent wheezing in children up until 3 years of age compared with no maternal SHS exposure. Similarly, Jedrychowski et al. reported that prenatal maternal exposure to SHS was significantly associated with persistent wheezing, which developed during the first year of life and was still present in the second year [29]. In contrast, Chen et al. reported that prenatal maternal exposure to SHS was not a significant risk factor for any wheezing phenotype in children between 0 and 9 years of age [30]. Thus, the available reports on the association between prenatal maternal exposure to SHS and children’s wheezing phenotypes are inconsistent. The difference in the results might be explained by differences in the SHS parameters assessed, including the number of cigarettes smoked, frequency of exposure, and timing of exposure.

Postnatal exposure of the infant to tobacco smoke outdoors was significantly associated with early transient and persistent wheezing compared with no postnatal SHS exposure, unlike indoor exposure. Jedrychowski et al. reported that postnatal exposure of the infant to SHS was not associated with any wheezing phenotypes up to the age of 2 years [29]. In a Japanese hospital-based birth cohort study involving children up to 9 years of age, postnatal exposure of the child to SHS was associated with only transient early wheezing, characterized by wheezing that peaked at 1–2 years of age and almost disappeared after the age of 5 [31]. Although the association between postnatal exposure of the child to SHS and wheezing phenotypes in early childhood is not consistent, there is a report of significant associations with wheezing onset at age ≥ 5 years [32], and the long-term effects need to be examined.

Unlike early transient and persistent wheezing, there was no dose-dependent association between the number of cigarettes smoked by the mother during pregnancy and late-onset wheezing. Several studies have reported that predisposition to atopy in children (eg, eczema), sensitization to food and aeroallergens, and a family history of asthma were associated with an increased risk of this wheezing phenotype [9, 31–34]. The clear differences between the risk factors for late-onset wheezing and other phenotypes may imply a different underlying pathophysiology.

This large-scale prospective cohort study used data obtained from across Japan to investigate the association of exposure to tobacco smoke from preconception to the postnatal period with wheezing phenotypes in children before the age of 3 years. In addition, we separately evaluated maternal smoking status during the preconception, prenatal, and postnatal periods, maternal exposure to SHS during pregnancy, and postnatal exposure of the infant to SHS. In the analysis, we also controlled for many potential covariates, such as demographic variables, lifestyle factors, and medical history. Our findings emphasize the importance of interventions minimizing tobacco smoke exposure associated with these wheezing phenotypes in children. Therefore, relevant strategies are needed to promote smoking cessation from any source by the mother and those around her during the preconception, prenatal, and postnatal periods.

Our study had a few limitations. First, longitudinal studies are at risk of bias due to missing data, such as incomplete data acquisition and loss of follow-up due to their design. Second, we assessed smoking status (prenatal or postnatal exposure to active smoking or SHS) and wheezing episodes via self-administered questionnaires, which may have resulted in under-reporting and recall bias. Third, only about half of the eligible fathers participated and only the mothers were asked about their SHS exposure status. All of these factors might have affected the results.

## Conclusions

In conclusion, early transient and persistent wheezing were associated with maternal smoking before and throughout pregnancy and with prenatal maternal exposure to SHS. Therefore, in addition to promoting smoking cessation among pregnant women, interventions and policies are needed to more broadly encourage smoking cessation, including among prepregnant women and family members living together.

## Supplementary Information

Additional file 1: Supplementary Table 1. Questionnaire Content.

Additional file 2: Supplementary Table 2. Children with history of allergic disease.

Additional file 3: Supplementary Table 3. Associations between tobacco smoke exposure and wheezing phenotypes only among children with no history of allergic disease (adjusted models).

## Data Availability

Data are unsuitable for public deposition due to ethical restrictions and the legal framework of Japan. It is prohibited by the Act on the Protection of Personal Information (Act No. 57 of 30 May 2003, amendment 9 September 2015) to publicly deposit data containing personal information. Ethical Guidelines for Medical and Health Research Involving Human Subjects enforced by the Japan Ministry of Education, Culture, Sports, Science, and Technology and the Ministry of Health, Labour and Welfare also restrict the open sharing of epidemiological data. All inquiries about access to data should be sent to: jecs-en@nies.go.jp. The person responsible for handling enquiries sent to this e-mail address is Dr. Shoji F. Nakayama, JECS Programme Office, National Institute for Environmental Studies.

## Abbreviations

aOR: Adjusted odds ratio
CI: Confidence interval
DOHaD: Developmental Origins of Health and Disease
JECS: Japan Environment and Children’s Study
OR: Odds ratio
SHS: Secondhand smoke
