# Supplementary material for: Maternal exposure to smoking and wheezing phenotypes in children: a cohort study of the Japan Environment and Children’s Study
Source: BMC Pediatr. 2024 Oct 1;24:624. doi: 10.1186/s12887-024-05101-6 (PMC11443675; doi:10.1186/s12887-024-05101-6)
Supplement: Supplementary file 1 — Additional file 1: Supplementary Table 1. Questionnaire Content. [file 12887_2024_5101_MOESM1_ESM.docx]

**Supplementary Materials**

**Additional file 1: Supplementary Table 1.** Questionnaire Content.

| Variables | Respondents | Response period | Question | Answer |
| --- | --- | --- | --- | --- |
| Maternal smoking status | Mother | When the children was 1 month of age | Smoking history | 1 = Never,  2 = Previously did, but quit before recognizing current pregnancy,  3 = Previously did, but quit after finding out current pregnancy,  4 = Yes, I still smoke 1–10 cigarettes/day,  5 = Yes, I still smoke 11–20 cigarettes/day, 6 = Yes, I still smoke ≥ 21 cigarettes/day |
| Prenatal maternal exposure to SHS | Mother | In second/third trimester | The number of days per week of exposure to tobacco smoke at home, in the workplace, or in other indoor locations | 1 = Almost never,  2 = ≤ 1 day per week,  3 = 2–3 days per week,  4 = 4–6 days per week,  5 = Every day |
| Postnatal exposure of the infant to SHS | Mother | When the children was 1 month of age | Location of exposure to tobacco smoke | 1 = None, 2 = Outdoor, 3= Indoor |
| Wheezing | Caregivers | When the children was 1 year of age | Has your child ever had wheezing or whistling in the chest in the last 12 months? | 0 = No, 1 = Yes |
| Wheezing | Caregivers | When the children was 3 year of age | Has your child ever had wheezing or whistling in the chest in the last 12 months? | 0 = No, 1 = Yes |
| Maternal age at delivery | Doctor | At birth | Maternal age | |
| Maternal BMI before pregnancy | Doctor | In first trimester | Calculated from the height and weight. | |
| Physical activity during mid-late pregnancy | Mother | In second/third trimester | International Physical Activity Questionnaire (IPAQ) short form | A response of "Yes" to any of the items asking about walking, moderate or high physical activity on an ordinary day in a week |
| Marital status | Mother | In first trimester | Current marital status | 1 = Married (including common-low marriage),  2 = Single (Never married) ,  3 = Divorced,  4 = Widowed |
| Maternal education | Mother | In second/third trimester | Highest level of education | 1 = Junior high school,  2 = High school,  3 = Technical junior college,  4 = Technical/vocational college,  5 = Associate degree,  6 = Bachelor’s degree,  7 = Graduate degree (Master’s/Doctor’s) |
| Maternal employment status | Mother | When the children was 1 year of age | Current employment status | 1=Permanent full-time employee,  2=Self-employed,  3=Temporary full-time employee,  4=Full-time homemaker or leave of absence,  5=Part-time employee,  6=Unemployed, 7=Other |
| Maternal alcohol consumption | Mother | In second/third trimester | Alcohol intake | 1=Never,  2=Previously did, but quit before learning of current pregnancy,  3=Previously did, but quit after learning of current pregnancy,  4=Yes, I still drink |
| Maternal history of allergy | Mother | In first trimester | Maternal history of allergy (asthma, atopic dermatitis, food allergy, allergic rhinitis, or allergic conjunctivitis) based on a physician's diagnosis | |
| Sex | Doctor | At birth | Sex | |
| Gestational weeks | Doctor | At birth | Gestational weeks | |
| Birth weight | Doctor | At birth | Weight at birth(g) | |
| Type of delivery | Doctor | At birth | Mode of delivery | 1 = Spontaneous delivery,  2 = Induced delivery,  3 = Vacuum extraction,  4 = Forceps delivery,  5 = Planned Cesarean delivery/Emergent Cesarean delivery |
| Birth season | Doctor | At birth | Birth season | |
| Parity | Doctor | In first trimester | Parity | |
| Infant anomalies | Doctor | At birth | Physical anomalies | 1 = No, 2 = Yes |
| Daycare attendance | Caregivers | When the children was 6 months of age | Daycare attendance | 1 = No, 2 = Yes |
| Pet ownership | Caregivers | When the children was 6 months of age | Having pet(s) at home | 1 = No, 2 = Yes |
| Household income, million yen/year | Mother | In second/third trimester | Annual household income | 1 = Less than 2 million yen,  2 = 2 million yen to less than 4 million yen,  3 = 4 million yen to less than 6 million yen,  4 = 6 million yen to less than 8 million yen,  5 = 8 million yen to less than 10 million yen,  6 = 10 million yen to less than 12 million yen,  7 = 12 million yen to less than 15 million yen,  8 = 15 million yen to less than 20 million yen,  9 = 20 million yen or over |
| Abbreviation: SHS, secondhand smoke; BMI, body mass index; IPAQ, International Physical Activity Questionnaire. | | | | |
