# Supplementary material for: Maternal exposure to smoking and wheezing phenotypes in children: a cohort study of the Japan Environment and Children’s Study
Source: BMC Pediatr. 2024 Oct 1;24:624. doi: 10.1186/s12887-024-05101-6 (PMC11443675; doi:10.1186/s12887-024-05101-6)
Supplement: Supplementary file 2 — Additional file 2: Supplementary Table 2. Children with history of allergic disease. [file 12887_2024_5101_MOESM2_ESM.docx]

**Supplementary Materials**

**Additional file 2: Supplementary Table 2.** Children with history of allergic disease.

|  | 1 year | | 1.5 years | | 2 years | | 3 years | |
| --- | --- | --- | --- | --- | --- | --- | --- | --- |
|  | 73057 | | 71915 | | 71590 | | 73057 | |
| Children with history of allergic disease | | | | | | | | |
| Atopic dermatitis | 3078 | (4.2%) | 4466 | (6.2%) | 4138 | (5.8%) | 5326 | (7.3%) |
| Food allergy | 4850 | (6.6%) | 7316 | (10.2%) | 5118 | (7.2%) | 4636 | (6.4%) |
| Allergic rhinitis,  Allergic conjunctivitis | 872 | (1.2%) | 1905 | (2.7%) | 2217 | (3.1%) | 4028 | (5.5%) |
