# Supplementary material for: Maternal exposure to smoking and wheezing phenotypes in children: a cohort study of the Japan Environment and Children’s Study
Source: BMC Pediatr. 2024 Oct 1;24:624. doi: 10.1186/s12887-024-05101-6 (PMC11443675; doi:10.1186/s12887-024-05101-6)
Supplement: Supplementary file 3 — Additional file 3: Supplementary Table 3. Associations between tobacco smoke exposure and wheezing phenotypes only among children with no history of allergic disease (adjusted models). [file 12887_2024_5101_MOESM3_ESM.docx]

| **Supplementary Materials**  **Additional file 3: Supplementary Table 3.** Associations between tobacco smoke exposure and wheezing phenotypes only among children with no history of allergic disease (adjusted models) | | | | | | | | | |
| --- | --- | --- | --- | --- | --- | --- | --- | --- | --- |
|  | Child's wheezing phenotype during the first 3 years of life | | | | | | | | |
|  | Early transient wheezing | | | Late-onset wheezing | | | Persistent wheezing | | |
|  | Cases/Subtotal | aOR^a^(95% CI) | p | Cases/Subtotal | aOR^a^(95% CI) | p | Cases/Subtotal | aOR^a^(95% CI) | p |
| Maternal smoking status at 1 month postpartum | | | | | | | | | |
| Never smoker | 2637/28447 | 1.00 |  | 2662/28472 | 1.00 |  | 1263/27073 | 1.00 |  |
| Quit smoking before learning of pregnancy | 1180/10234 | 1.14(1.05–1.23) | <0.001 | 1038/10092 | 1.10(1.01–1.19) | 0.024 | 565/9619 | 1.10(0.99–1.23) | 0.070 |
| Quit smoking after learning of pregnancy | 689/5812 | 1.17(1.06–1.29) | 0.002 | 601/5724 | 1.14(1.03–1.26) | 0.014 | 332/5455 | 1.15(1.01–1.33) | 0.041 |
| Current smoker during pregnancy |  |  |  |  |  |  |  |  |  |
| 1–10 cigarettes/day | 189/1122 | 1.43(1.20–1.71) | <0.001 | 111/1044 | 1.13(0.91–1.39) | 0.281 | 106/1039 | 1.52(1.21–1.93) | <0.001 |
| ≥ 11 cigarettes/day | 50/240 | 1.81(1.30–2.53) | <0.001 | 22/212 | 1.12(0.71–1.77) | 0.613 | 26/216 | 1.88(1.21–2.92) | 0.005 |
| p value for trend |  |  | <0.001 |  |  | 0.006 |  |  | <0.001 |
| Frequency of SHS exposure in second/third trimester | | | | | | | | | |
| Almost never | 2766/29958 | 1.00 |  | 2843/30035 | 1.00 |  | 1293/28485 | 1.00 |  |
| ≤ 1 day per week | 641/5402 | 1.25(1.13–1.37) | <0.001 | 535/5296 | 1.04(0.94–1.15) | 0.440 | 290/5051 | 1.17(1.03–1.34) | 0.020 |
| 2–3 days per week | 415/3481 | 1.15(1.03–1.29) | 0.016 | 327/3393 | 0.97(0.85–1.10) | 0.602 | 217/3283 | 1.29(1.11–1.51) | 0.001 |
| 4–6 days per week | 282/2117 | 1.22(1.07–1.41) | 0.004 | 231/2066 | 1.11(0.96–1.29) | 0.155 | 137/1972 | 1.22(1.01–1.48) | 0.042 |
| Every day | 641/4897 | 1.10(0.99–1.23) | 0.074 | 498/4754 | 1.05(0.94–1.18) | 0.397 | 355/4611 | 1.27(1.10–1.47) | 0.001 |
| p value for trend |  |  | 0.004 |  |  | 0.280 |  |  | <0.001 |
| Location of exposure to tobacco smoke at 1 month postpartum | | | | | | | | | |
| None | 2117/23181 | 1.00 |  | 2178/23242 | 1.00 |  | 982/22046 | 1.00 |  |
| Outdoor | 2486/21748 | 1.09(1.02–1.17) | 0.015 | 2162/21424 | 1.06(0.99–1.13) | 0.126 | 1224/20486 | 1.16(1.05–1.28) | 0.003 |
| Indoor | 142/926 | 1.22(1.00–1.49) | 0.051 | 94/878 | 1.09(0.87–1.37) | 0.471 | 86/870 | 1.54(1.19–1.99) | <0.001 |
| p value for trend |  |  | 0.006 |  |  | 0.118 |  |  | <0.001 |
| **Notes:** ^a^Adjusted for maternal age at delivery, maternal body mass index before pregnancy, maternal physical activity during mid-late pregnancy, marital status, maternal employment status, maternal education level, maternal alcohol consumption, maternal history of allergy, infant sex, gestational weeks at delivery, birth weight, mode of delivery, birth season, parity, infant anomalies, daycare attendance, pet ownership, and annual household income. | | | | | | | | | |
| **Abbreviations:** SHS, secondhand smoke; aOR, adjusted odds ratio; CI, confidence interval. | | | | | | | | | |
